# ﻿A new species of *Xenophrys* (Amphibia, Anura, Megophryidae) from southern Tibet, China

**DOI:** 10.3897/zookeys.1182.106828

**Published:** 2023-10-20

**Authors:** Guocheng Shu, Ke Li, Yayong Wu, Qin Liu, Zhongping He, Ling Li, He Zhang, Peng Guo

**Affiliations:** 1 Faculty of Agriculture, Forest and Food Engineering, Yibin University, Yibin 644007, China Yibin University Yibin China; 2 Key Lab of Aromatic Plant Resources Exploitation and Utilization in Sichuan Higher Education, Yibin 644007, China Key Lab of Aromatic Plant Resources Exploitation and Utilization in Sichuan Higher Education Yibin China; 3 College of Life Sciences, Shenyang Normal University, Shenyang 110034, China Shenyang Normal University Shenyang China

**Keywords:** Morphology, phylogenetic analyses, taxonomy, *Xenophryspangdaensis* sp. nov., Yadong County

## Abstract

A new species of *Xenophrys* is described from Yadong County, Tibet Autonomous Region, China based on morphological and molecular evidence. Phylogenetic analyses based on the mitochondrial genes 16S rRNA and COI indicated that this new species represents an independent lineage and the minimum *p*-distance based on 16S rRNA between this species and its congeners is 4.4%. Additionally, the new species is distinguished from its congeners by a combination of the following morphological characters: (1) small body size, SVL 17.9–22.2 mm in adult males and SVL 23.4 mm in the single adult female; (2) tympanum indistinct, supratympanic fold distinct; (3) canthus rostralis well-developed, snout tip far beyond the margin of the lower lip; (5) pupil vertical; (6) vomerine teeth present, maxillary teeth present; (7) tongue notched posteriorly; (8) supernumerary tubercles absent, subarticular, metacarpal and metatarsal tubercles indistinct; (9) relative finger lengths I < II < IV < III, finger tips rounded, slightly expanded relative to digit widths; (10) toes with narrow lateral fringes and tarsal folds; (11) a dark triangular marking with light edge between eyes, a dark “)(”-shaped marking, with light edge, present on center of dorsum, pectoral glands on sides of the breast.

## ﻿Introduction

The Asian horned toad Megophryinae are widely distributed from northern India (west of Nepal) to eastern China and south to the Sundas and the Philippines (Frost 2023). Currently, 132 species have been described until July 2023, more than half of which have been named since the turn of the century (Frost 2023). There has been controversy about the generic classification of this group for a long time (e.g., Huang and Fei 1981; Tian and Hu 1983; Dubois 1987; Lathrop 1997; Rao and Yang 1997; Jiang et al. 2003; Delorme et al. 2006; Fei et al. 2009; Fei and Ye 2016; Chen et al. 2017; Mahony et al. 2017; Liu et al. 2018; Dubois et al. 2021; Lyu et al. 2021, 2023; Frost 2023). So far, the Megophryinae were defined as comprising ten clades by recent multilocus phylogenetic studies, including *Atympanophrys*, *Brachytarsophrys*, *Megophrys*, *Ophryophryne*, *Boulenophrys*, *Pelobatrachus*, *Grillitschia*, *Jingophrys*, *Sarawakiphrys*, and *Xenophrys* (Lyu et al. 2023). Previously, most authors regarded these clades as five or seven genera (Chen et al. 2017; Liu et al. 2018), while few researchers held a conservative attitude and regarded them as seven subgenera (Mahony et al. 2017; Shi et al. 2020). The genus *Panophrys* was established by Rao and Yang (1997); however, Dubois et al. (2021) noted that the generic name was preoccupied by *Panophrys* Dujardin, 1840 (Protozoa), so they chose *Boulenophrys* to replace it under the Principle of Homonymy.

In this study, we followed the classification system in Dubois et al. (2021) and Lyu et al. (2023) that Megophryidae contains ten genera (*Atympanophrys*, *Brachytarsophrys*, *Megophrys*, *Ophryophryne*, *Boulenophrys*, *Pelobatrachus*, *Grillitschia*, *Jingophrys*, *Sarawakiphrys*, and *Xenophrys*). Currently, the genus *Xenophrys* contains 28 recognized species in the world, which are distributed in Nepal, Bhutan, Bangladesh, India, Myanmar, Thailand, Cambodia, Vietnam, Cambodia, China, and Malaysia, of which ten are recorded in China (Frost 2023). In fact, nearly one third of the species in the genus were described in the last five years (Mahony et al. 2018, 2020; Shi et al. 2020; Luong et al. 2022), so the species diversity of the genus may have been underestimated. The eastern Himalaya is one of the 36 global biodiversity hotspots (Basnet et al. 2019), many new species have been discovered and named in this region in recent years (e.g., Jiang et al. 2016a, b, c; Shi et al. 2020). Over the past two years, several field surveys were conducted in this area and specimens of the family Megophryidae were collected. Also, a new bush frog, *Raorchestesyadongensis* Zhang, Shu, Liu, Dong, & Guo, 2022, was recently found and described (Zhang et al. 2022). Based on morphological comparison and molecular phylogenetic analyses, some specimens were identified a new member of *Xenophrys*.

## ﻿Materials and methods

### ﻿Sampling

**Figure 1.**
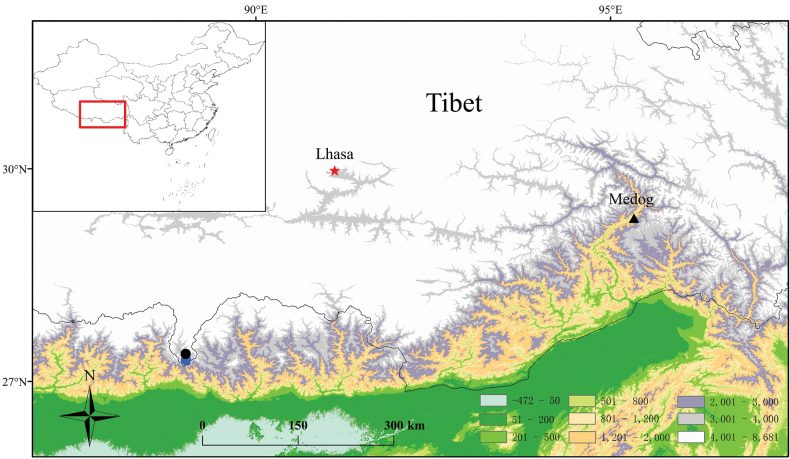
Type locality of the new species *Xenophrys* in Yadong County, Tibet Autonomous Region, China. The red star indicates the provincial capital, the black triangle indicates Medog County, the blue and black spots indicate the type locality and collection site of some tadpoles, respectively.

Field surveys were conducted in August 2020 and July 2021. In total, seven adult specimens of *Xenophrys* were collected from two sites in Yadong County, Tibet Autonomous Region, China (Fig. 1). Thirteen tadpoles of the new taxon were also collected in a puddle where the new taxon was found. In the field, after taking photographs, the toads were euthanized using isoflurane, and then specimens were fixed in 75% ethanol. Tissue samples were taken and preserved separately in 95% ethanol prior to fixation. Specimens collected in this work were deposited in Yibin University. The Animal Care and Use Committee of Yibin University provided full approval for this research (No. 202003).

### ﻿Molecular phylogenetic analysis

Total genomic DNA was extracted using TIANamp Genomic DNA Purification Kit (TIANGEN Bio-tech Co., Ltd., Beijing, China), following manufacturer instructions. Two fragments of mitochondrial genes, 16S ribosomal RNA gene (16S) and the cytochrome C oxidase 1 gene (COI), were amplified and sequenced. Primer sequences were retrieved from the literature for 16S (Simon et al. 1994) and COI (Che et al. 2011), respectively. PCR amplifications were performed in a 25 μl volume reaction with the following conditions: an initial denaturing step at 95 °C for 4 min; 36 cycles of denaturing at 95 °C for 40 s, annealing at 55 °C (for 16S)/52 °C (for COI) for 40 s and extending at 72 °C for 70 s, and a final extending step of 72 °C for 10 min. PCR products were sequenced with both forward and reverse primers same as used in PCR. Sequencing was conducted using an ABI3730 automated DNA sequencer in Sangon Biotechnologies Co., Ltd. (Shanghai, China). New sequences were uploaded to GenBank (for accession numbers see Table 1).

**Table 1.** Information of samples used in the molecular analyses.

Species	Voucher ID	16S	COI	References
*A.gigantica*	SYS a003934	MH406766	MH406225	Liu et al. 2018
*A.shapingensis*	SYS a005310; KIZ YPX37515	MH406890	MH406352	Liu et al. 2018
*A.wawuensis*	SYS a005311	MH406891	MH406353	Liu et al. 2018
*Bo.binglingensis*	SYS a005313; KIZ 025807; FMNH 232874	MH406892	MH406354	Liu et al. 2018; Chen et al. 2017; Mahony et al. 2017
*Bo.boettgeri*	SYS a004149; KIZ YPXJK033	MF667878	MH406247	Liu et al. 2018; Chen et al. 2017
*Bo.brachykolos*	SYS a002258; ROM 16634	KJ560403	MH406120	Liu et al. 2018; Chen et al. 2017
*Bo.cheni*	SYS a004050	MF667873	MH406241	Liu et al. 2018
*Bo.chishuiensis*	SYS a005307; KIZ 025788	MH406888	MH406350	Liu et al. 2018; Chen et al. 2017
*Bo.daoji*	SYS a004089	MH406783	MH406243	Liu et al. 2018
*Bo.fansipanensis*	VNMN 2018.01	MH514886	MW086544	Tapley et al. 2021
*Bo.frigida*	AMS R186131	MT364279	MW086550	Tapley et al. 2021
*Bo.huangshanensis*	SYS a002702; KIZ 022004	MF667882	MH406160	Liu et al. 2018; Chen et al. 2017
*Bo.kuatunensis*	SYS a003449	MF667881	MH406206	Liu et al. 2018
*Bo.minor*	SYS a003212; KIZ YPX37545	MF667865	MH406197	Liu et al. 2018; Chen et al. 2017
*Bo.nanlingensis*	SYS a001962	MH406645	MH406081	Liu et al. 2018
*Bo.sangzhiensis*	SYS a004313; KIZ YPX11006	MH406802	MH406264	Liu et al. 2018; Chen et al. 2017
*Bo.spinata*	SYS a002226; KIZ 016100	MH406675	MH406115	Liu et al. 2018; Chen et al. 2017
*Bo.wushanensis*	SYS a003009; KIZ 045469	MH406733	MH406185	Liu et al. 2018; Chen et al. 2017
*Br.chuannanensis*	SYS a004927	MH406902	MH406365	Liu et al. 2018
*Br.feae*	SYS a003913; KIZ 046706	MH406900	MH406363	Liu et al. 2018; Chen et al. 2017
*Br.orientalis*	SYS a004225	OQ180989	MT162625	Li et al. 2020; Lyu et al.2023
*Br.platyparietus*	SYS a005919	OQ180990	MT162633	Li et al. 2020; Lyu et al. 2023
*Br.popei*	SYS a001864	KM504256	MH406361	Liu et al. 2018
*G.aceras*	LSUHC 7038	GQ995534	N	Chen et al. 2017
*G.longipes*	IABHU 21101	AB530656	N	Hasan et al. 2014
J.cf.pachyproctus	CIB022017061805	MN963228	MN964303	Shi et al. 2020
*J.vegrandis*	Z11605 HT	KY022305	MH647530	Mahony et al. 2020
*J.yeae*	CIB201706MT02	MN963216	MN964313	Shi et al.2020
*J.zhoui*	CIBMT171053	MN963207	MN964322	Shi et al.2020
*J.feii*	SYS a003876	OQ181007	OQ180893	Lyu et al. 2023
*Lep.alpina*	SYS a003927	MH406905	MH406368	Liu et al. 2018
*Lep.laui*	SYS a003471	MH406903	MH406366	Liu et al. 2018
*M.acehensis*	MZB Amph 26098	MT710708	N	Munir et al. 2021
*M.lancip*	ENS 7577	KX773567	N	Mahony et al. 2017
*M.montana*	LSUMZ 81916; UTA A-53736, ENS 7381	KX811927	KX812163	Chen et al. 2017; Mahony et al. 2017
*M.parallela*	RMAS 022	KY679898	N	Munir et al. 2018
*M.selatanensis*	MZB Amph 22411	MT710704	N	Munir et al. 2021
*O.hansi*	AMNH 169285	KY022204	KX812155	Mahony et al. 2017; Chen et al. 2017
*O.microstoma*	AMNH 168682	KY022199	N	Mahony et al. 2017
*O.poilani*	AMNH 169287	KY022202	N	Mahony et al. 2017
*P.baluensis*	IRSNB 15926	DQ642121	N	Mahony et al. 2017
*P.edwardinae*	FMNH 273694	KX811918	KX812050	Chen et al. 2017
*P.kalimantanensis*	KUHE 53577	AB719248	N	Hamidy et al. 2012
*P.kobayashii*	UNIMAS 8148	KJ831313	N	Oberhummer et al. 2014
*P.ligayae*	KUKUH309095; ZMMUNAP-05015	KY022192	KX812051	Mahony et al. 2017; Chen et al. 2017
*P.stejnegeri*	FMNH 250842; KU 314303	KY022190	KX812052	Mahony et al. 2017; Chen et al. 2017
*S.dringi*	UNIMAS 8948	KJ831316	N	Oberhummer et al. 2014
*X.ancrae*	Z11606 [S2011.307] HT	MN734391	N	Mahony et al. 2020
*X.auralensis*	NCSM 79599	KX811807	N	Mahony et al. 2018
*X.awuh*	BN6069 PT	KY022319	N	Mahony et al. 2020
*X.dzukou*	BN6072 HT	KY022324	N	Mahony et al. 2020
*X.flavipunctata*	SDBDU 2009.297 TT	KY022307	MH647536	Mahony et al. 2018
*X.glandulosa*	SYSa003795	MH406760	MH406219	Shi et al. 2021
*X.himalayana*	BNHS 6050	MH647526	N	Mahony et al. 2018
*X.lekaguli*	FMNH 265955 PT	KY022214	N	Mahony et al. 2017
*X.major*	SDBDU 2007.229	MH647514	N	Mahony et al. 2018
*X.mangshanensis*	KIZ021786	KX811790	KX812079	Shi et al. 2020
*X.maosonensis*	ROM 16679	KX811784	KX812081	Shi et al.2020
*X.medogensis*	CIB022017062002	MN963219	MN964310	Shi et al.2020
*X.megacephala*	ZSIC A 11213 HT	KY022315	MH647533	Mahony et al. 2018
*X.monticola*	SDBDU 2011.1047	KY022312	N	Mahony et al. 2017
*X.numhbumaeng*	BN6076 PT	MN734393	N	Mahony et al. 2020
*X.oreocrypta*	BN6046 PT	KY022306	N	Mahony et al. 2020
*X.oropedion*	SDBDU 2009.299	KY022317	MH647534	Mahony et al. 2018
*X.pangdaensis* sp. nov.	YBU21248 HT	OR026569	OR026034	This study
*X.pangdaensis* sp. nov.	YBU21259 PT	OR026570	OR026035	This study
*X.pangdaensis* sp. nov.	YBU21260 PT	OR026571	OR026036	This study
*X.pangdaensis* sp. nov.	YBU21261 PT	OR026572	OR026037	This study
*X.pangdaensis* sp. nov.	YBU21262 PT	OR026573	OR026038	This study
*X.pangdaensis* sp. nov.	YBU21269 PT	OR026574	OR026039	This study
*X.periosa*	BNHS 6061 PT	KY022309	MH647528	Mahony et al. 2018
*X.robusta*	SDBDU 2011.1057 TT	KY022314	MH647535	Mahony et al. 2018
*X.serchhipii*	SDBDU 2009.612	KY022323	MH647532	Mahony et al. 2018
*X.takensis*	FMNH 261711	KY022215	N	Mahony et al. 2017
*X.truongsonensis*	IEBR A.4952	ON146202	N	Luong et al. 2022
*X.zhangi*	KIZ014278	KX811765	KX812084	Mahony et al. 2018
*X.zunhebotoensis*	RGK 0041 TT	KY022322	N	Mahony et al. 2018,
*X.dehongensis*	SYS a003443; KIZ 048507	MH406746	MH406204	Liu et al. 2018; Chen et al. 2017
*X.katabhako*	K5204/ZSI 11401 HT	KX894667	N	Deuti et al. 2017
*X.lancangica*	SYS a002961; KIZ01464; AMNH168679	MH406728	MH406180	Liu et al. 2018; Chen et al. 2017; Mahony et al. 2017
*X.parva*	SYS a003042; KIZ YPX27643	MH406737	MH406189	Liu et al. 2018; Chen et al. 2017
*X.sanu*	K5197/ZSI 11392 HT	KX894678	N	Deuti et al. 2017

Specimen status: HT, holotype; PT, paratype; TT, topotype.

For molecular analyses, the available sequences of *Xenophrys* species were downloaded from GenBank, especially for their holotypes and/or topotypes for which comparable sequences were available (Table 1). Representative species sequences for all recognized megophryid genera were also downloaded for phylogenetic analysis (also including two controversial species *X.katabhako* comb. nov. and *X.sanu* comb. nov.). Sequences were assembled and aligned using the Clustalw module in BioEdit 7.0.9.0 (Hall 1999) with default settings. Alignments were checked by eye and revised manually if necessary. PartitionFinder v. 2.1.1 (Lanfear et al. 2017) was used to select the corresponding best-fit nucleotide substitution models for 16S gene/each codon position of COI gene under the Akaike Information Criteria (AIC). Phylogenetic analyses of the concatenated-sequence matrix were conducted in MrBayes v. 3.2.4 (Ronquist et al. 2012). Two independent runs were conducted in the BI analysis, and each run consisted of 5 × 10^7^ generations, sampled every 1000 generations. Runs were considered to have converged when the average standard deviation of split frequencies (ASDSF) was less than 0.01. The first 25% of generations were removed as the “burn-in” stage followed by calculation of Bayesian posterior probabilities (BPP) and the 50% majority-rule consensus of the post burn-in trees sampled at stationarity. The phylogenetic trees were visualized using FigTree 1.4.3 (Rambaut 2016). Mean genetic distances between *Xenophrys* species were calculated in MEGA 7 (Kumar et al. 2016) using the uncorrected p-distance model based on 16S gene (some species lack of COI gene).

### ﻿Morphological analysis

A total of seven adult specimens were measured. The terminology and methods followed Mahony (2011). Measurements were taken with a dial caliper to the nearest 0.1 mm. Thirty characters of adult specimens were measured:

**EL** eye length (horizontal distance between the anterior and posterior borders of orbit);

**EN** eye-nostril length (distance from front of eye to the center of nostril);

**FAL** forearm length (distance from elbow to wrist);

**FIL** first finger length (distance from the tip of the first digit to its base where it joins the second digit);

**FIIL** second finger length (distance from the tip of the second digit to its base where it joins the first digit);

**FIIIL** third finger length (distance from the tip of the third digit to its base where it joins the second digit);

**FIVL** fourth finger length (measured from the tip of the fourth digit to its base where it joins the third digit);

**FIIIW** minimum third finger width (taken at the base of the terminal portion of the digit, which is expanded on some species);

**FIIIDW** maximum width of the third fingertip;

**FOL** foot length (distance from the proximal end of the inner metatarsal tubercle to the tip of the fourth digit);

**HAL** hand length (distance from wrist to tip of third digit);

**HL** head length (distance from the rear of the mandible to the tip of the snout);

**HLL** hindlimb length;

**HW** head width (distance between the posterior angles of jaw);

**IBE** internal back of eyes (the shortest distance between the posterior borders of the orbits);

**IFE** internal front of eyes (shortest distance between the anterior borders of orbits);

**IMT** length of the inner metatarsal tubercle;

**IN** internarial distance (shortest distance between two nostrils);

**IUE** inter upper eyelid width (shortest distance between upper eyelids);

**SHL** shank length (distance from knee to ankle);

**SL** snout length (distance from tip of snout to anterior border of the orbit);

**SN** nostril-snout length (distance from center of the nostril to tip of the snout);

**SVL** snout-vent length (distance from the tip of the snout to the posterior edge of the vent);

**TFOL** tarsal-foot length (distance from heel to the tip of the fourth digit);

**TIVW** minimum fourth toe width (taken at the base of the terminal portion of the digit, which is expanded on some species);

**TIVDW** maximum width of the fourth toe tip;

**TL** thigh length (distance from cloaca to knee);

**TYD** largest tympanum diameter;

**TYE** tympanum-eye distance (distance from the anterior border of the tympanum to the posterior orbital border);

**UEW** maximum upper eyelid width.

Thirteen tadpoles of the new taxon were measured. The stages of tadpoles were identified following Gosner (1960). Seventeen morphometric characters of tadpoles were measured:

**BH** maximum body height;

**BL** body length (distance from tip of snout to trunk-tail junction);

**BW** maximum body width;

**ED** maximum eye diameter;

**IND** internasal distance (distance between center of two naris);

**LF** maximum height of lower tail fin;

**NE** naris-eye distance (distance from center of naris to anterior corner of eye);

**ODW** oral disc width (largest width of oral disc);

**PP** interpupilar distance;

**RN** rostro-narial distance (distance from tip of snout to center of naris);

**SS** snout-spiracle distance (distance from tip of snout to opening of spiracle);

**SU** snout-upper fin distance (distance from snout to beginning of upper tail fin);

**TAL** tail length (distance between posterior side of opening of cloaca to tip of tail);

**TMH** maximum tail muscle height;

**TMW** maximum tail muscle width;

**TOL** total length;

**UF** maximum height of upper tail fin.

Sex and maturity of the specimens were confirmed by direct examination of secondary sexual characters, including vocal sacs, nuptial pads, and the gonadal inspection (Fei and Ye 2016; Mahony et al. 2020). For webbing description, we followed Glaw and Vences (2007). We compared the morphological characters of the new species with literature data for 28 other species of *Xenophrys*.

## ﻿Results

### ﻿Phylogenetic analyses

**Figure 2.**
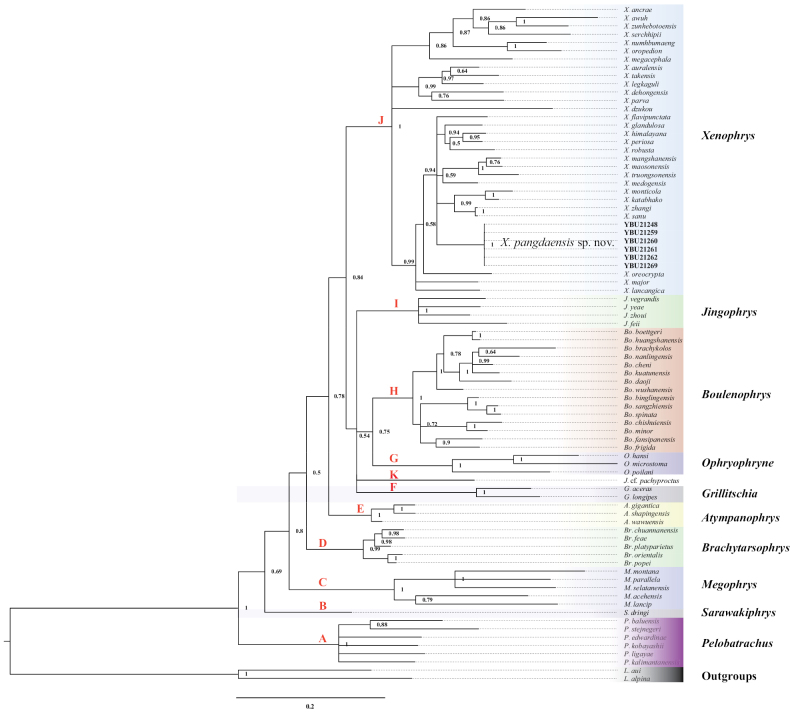
Phylogenetic tree of the genus *Xenophrys* inferred from two mitochondrial gene fragments by Bayesian inference.

The aligned sequence matrix of 16S and COI genes contained 574 bps and 663 bps, respectively. Except *X.damrei*, all other species of *Xenophrys* were included in the phylogenetic analysis. The model selection suggested that GTR+I+G as the best model for 16S rRNA fragment, and GTR+I+G, GTR+I+G, and HKY+I as the best model for the first, second and third codon position of COI gene, respectively. The BI phylogenetic tree is shown in Fig. 2 with Bayesian posterior probabilities (BPP) for major nodes. The phylogenetic tree showed that all *Xenophrys* species formed a monophyletic lineage containing 11 independent clades, though some relationships were not resolved in the tree. Those clades correspond exactly to the ten genera previously recognized, including *Pelobatrachus* (clade A), *Sarawakiphrys* (clade B), *Megophrys* (clade C), *Brachytarsophrys* (clade D), *Atympanophrys* (clade E), *Grillitschia* (clade F), *Ophryophryne* (clade G), *Boulenophrys* (clade H). *Jingophrys* (clade I), *Xenophrys* (clade J), and clade K. Significantly, J.cf.pachyproctus alone formed a clade. All samples from Yadong were strongly supported to be a monophyletic group and formed sister relationships with *X.flavipunctata*, *X.glandulosa*, *X.himalayana*, *X.periosa*, *X.robusta*, *X.mangshanensis*, *X.maosonensis*, *X.truongsonensis*, *X.medogensis*, *X.megdogensis*, *X.monticola*, and *X.zhangi*. *X.katabhako* comb. nov. and *X.monticola* formed a clade. *X.sanu* comb. nov. and *X.zhangi* clustered into another clade.

Interspecific uncorrected *p*-distance of the *Xenophrys* species ranged from 0.0 (*X.zhangi* and *X.sanu* comb. nov.) to 18.7% (*X.awuh* and *X.dzukou*) (Table 2). The minimum *p*-distance between the unidentified specimens and any other species of *Xenophrys* was 4.4% (with *X.glandulosa*) (Table 2).

**Table 2.** Uncorrected pairwise sequence divergences (*p*-distance, in %) based on 16S ribosomal RNA sequences.

No.	Species	1	2	3	4	5	6	7	8	9	10	11	12	13	14	15	16	17	18	19	20	21	22	23	24	25	26	27	28	29	30
1	*X.ancrae*																														
2	*X.auralensis*	9.4																													
3	*X.awuh*	9.9	14.7																												
4	*X.dzukou*	6.9	9	18.7																											
5	*X.flavipunctata*	8.5	9.4	13.6	10.2																										
6	*X.glandulosa*	7.7	7.1	12.1	7.5	4.6																									
7	*X.himalayana*	7.9	6.7	10.5	8.6	5.2	3.3																								
8	*X.legkaguli*	8.4	4	14.8	8.9	9.6	7.8	7.6																							
9	*X.major*	7.9	7.7	11.4	8.9	6.6	5.5	4.7	8.8																						
10	*X.mangshanensis*	8.2	8	11.5	9.7	5.7	5.6	5.7	9.3	6.9																					
11	*X.maosonensis*	7.9	8.4	12.4	9.7	6.1	5.6	5.2	9	6.5	1.9																				
12	*X.medogensis*	7	7.4	11.7	8.2	5	3.8	3.3	7.8	5.2	5.4	5																			
13	*X.monticola*	9.7	7.5	13.3	10.2	7	5.2	5.6	9	7.4	6.3	6.9	6.7																		
14	*X.megacephala*	7.1	9.5	12.1	8.2	9.8	9.1	8.6	8.6	8.4	8.7	8.9	9	10.2																	
15	*X.numhbumaeng*	8.2	10.2	10.7	12.2	10	9.8	10	11.5	9.8	10.7	10.9	9.5	11	7.8																
16	*X.oreocrypta*	9.1	8.6	14.5	10.2	7.4	6.9	6.6	8.4	7	8.4	7.8	7.5	8.5	9.4	10.5															
17	*X.oropedion*	8.5	11.1	13.4	11.8	11.3	10.7	10.5	11.2	10.5	10.1	10.7	10.5	11.3	7.8	5.1	11.1														
18	*X.periosa*	8.7	7.3	12.2	8.9	5.6	3.1	2.5	7.8	6	6.1	5.9	3.3	6.2	8.6	10.2	7.2	10.7													
19	*X.robusta*	9.9	8.8	13.3	11.5	6.2	4.4	4.6	9.2	6.8	6.9	6.9	4.8	6.2	10.4	10.6	7.8	11.9	4.1												
20	*X.serchhipii*	8.3	11.4	8.7	11.5	10.2	10.6	10	10.9	10	9.4	10.2	10.4	12.4	8.5	9.2	11.6	10.3	10.4	11.6											
21	*X.takensis*	9.5	4	14.2	9.9	9.4	7.2	6.8	4.7	8.4	8.2	8.4	6.7	8.6	8.8	10.9	9	11.4	7.4	8.6	10.8										
22	*X.truongsonensis*	8	7.7	12.4	10.6	7.2	6.3	6.5	7.9	6.5	3.6	3.3	5.2	7.8	9.1	10.7	7.7	10.4	7.2	7.2	9.9	9									
23	*X.zhangi*	7	6.5	11.4	9	5.6	3.8	4	7.4	4.8	5.2	5	3.6	4.4	9.3	8.9	7.3	10.5	4.4	4.8	10.6	6.7	5								
24	*X.zunhebotoensis*	7.2	12.2	8.1	10.2	10.7	9.9	10.4	12.2	10.6	10.8	11	9.7	11.7	9.2	8.3	11.6	10	10.9	11.9	8.3	11.4	10.9	10.3							
25	*X.katabhako*	8.4	9.5	12.9	9.6	7.3	4.9	5.9	9.3	7.1	5.7	6.3	5.9	2.7	10.1	11.9	8.9	12.5	6.8	6.2	12.2	8.7	7.4	4.3	11						
26	*X.lancangica*	7	7.8	13.6	8.2	7.7	5.9	6.7	7.6	6.9	8.1	7.7	6.7	9.2	8.8	10	7.7	11.3	7.7	7.5	10.8	8.2	7.5	7.1	10.5	8.1					
27	*X.sanu*	8.1	8.6	13.3	9.6	6.8	4.9	4.6	8.7	5.7	6.8	6.5	4.3	5.1	10.5	10.8	8.7	12.8	5.4	5.7	12.2	8.2	6.3	0	11.5	4.3	8.9				
28	*X.dehongensis*	9.9	6.4	16.3	10.1	9.7	7.1	8.2	7.4	9.1	9.3	9.9	8.3	9.7	10.2	11.6	8.5	12.7	8.4	8.4	13.5	7.2	9.5	7.3	13.2	10.4	7.9	9.3			
29	*X.parva*	10.5	6.7	15.9	10.8	10.1	7.5	7.8	7.4	8	8.9	9.1	7.7	9.2	10.2	12	8.6	12.3	8	8.2	12.2	7.4	8.4	7.5	12.7	9.9	7.5	9	6.8		
30	*X.pangdaensis* sp.nov.	7.3	6.5	12.5	9.5	6	4.4	5.6	7.4	6.2	6.1	5.7	4.6	6.2	9.8	10	7.4	11.3	5	5.2	11	7.6	6.1	4.6	10	6.5	5.9	6.3	8.3	7.7	

### ﻿Morphological analysis

All samples from Yadong shared many morphological characters with *Xenophrys* species, including dorsal skin texture basically smooth, vomerine teeth present, ventral colorations, lateral fringes and webbings on toes, tongues notched posteriorly, maxillary teeth present, and tympanum indistinct. However, they can be distinguished from all recognized congeners by a combination of distinctive morphological characters (see taxonomic accounts below) and these specimens are therefore described as a new species based on the phylogenetic analyses and morphological comparisons.

### ﻿Taxonomic accounts

#### ﻿ Xenophrys pangdaensis sp. nov.

Taxon classificationAnimaliaAnuraMegophryidae

54A0DCC4-A14C-54C0-90DB-0ED92995F026

https://zoobank.org/262319B9-D690-4FDB-9A44-621FD390956E

Fig. 3

##### Type material.

***Holotype*.** YBU21248, adult male, collected by Ke Li and He Zhang on 28 August 2021 from Pangda Village (27°17.25'N, 89°0.42'E; ca. 2000 m a.s.l.), Yadong Town, Yadong County, Tibet Autonomous Region, China.

***Paratypes*.** Six adult specimens (males: YBU21258, YBU21259, YBU21260, YBU21261, YBU21269; female: YBU21262) were collected from two very close sites in Yadong Town by Ke Li and He Zhang on 28 August 2021.

##### Other specimens examined.

Thirteen tadpoles were collected by Ke Li and He Zhang on 4 September 2021. Five tadpoles were collected from Pangda Village (17 km, 27°18.18'N, 89°0.34'E), Yadong Town. The other tadpoles were collected from Pangda Village (27°17.25'N, 89°0.42'E) together with the holotype.

##### Etymology.

The species name *pangdaensis* indicates the type locality of Pangda Village, Yadong County, Tibet Autonomous Region, China.

##### Suggested name.

Pangda Horned Toad (English), and Pang Da Jiao Chan (庞达角蟾, Chinese).

##### Diagnoses.

(1) Small body size, SVL 17.9–22.2 mm (20.5±1.8, *n* = 6) in adult males and SVL 23.4 mm (*n* = 1) in the adult female (Table 3); (2) tympanum indistinct, supratympanic fold distinct; (3) canthus rostralis well-developed, snout tip far beyond the margin of the lower lip; (5) pupil vertical; (6) vomerine teeth present, maxillary teeth present; (7) tongue notched terminally; (8) supernumerary tubercles absent, subarticular, metacarpal and metatarsal tubercles indistinct; (9) relative finger lengths I < II < IV < III, finger tips rounded, slightly expanded relative to digit widths; (10) toes with narrow lateral fringes and tarsal folds; (11) a dark triangular marking with light edge between eyes, a dark)(-shaped marking, with light edge, present on center of dorsum, pectoral glands on sides of the breast.

**Table 3.** Measurements (in mm) of the type series of *Xenophryspangdaensis* sp. nov.

	YBU21248 (holotype)	YBU21262 (paratype)	YBU21261 (paratype)	YBU21259 (paratype)	YBU21260 (paratype)	YBU21269 (paratype)	YBU21258 (paratype)
Sex	male	female	male	male	male	male	male
SVL	21.5	23.4	21.7	20.9	18.6	17.9	22.2
FAL	3.4	5.9	7.5	5.5	4.3	3.6	4.6
HAL	6.5	6.3	7.2	5.2	5.1	5.8	8.0
HLL	30.4	33.5	30.5	34.9	30.4	26.6	35.4
SHL	10.9	11.4	11.3	9.8	9.1	9.2	11.6
SL	3.3	3.0	3.1	2.6	2.7	1.8	3.0
FOL	10.8	10.1	11.2	9.7	6.5	8.9	10.9
IN	2.5	2.9	2.1	2.4	2.9	3.1	2.7
IUE	2.9	2.3	2.4	2.1	3.3	2.5	3.4
UEW	1.9	2.0	1.6	1.3	2.11	2.5	1.9
TYD	1.8	1.1	1.7	1.7	1.8	1.6	1.5
HL	7.6	6.7	7.6	6.3	5.4	6.2	7.4
HW	6.9	7.1	7.0	6.2	6.2	6.5	8.5
FIL	2.6	2.6	3.3	2.3	2.2	2.5	3.2
FIIL	3.6	3.9	3.9	3.4	2.6	2.8	3.3
FIIIL	6.0	5.9	5.4	4.8	4.8	5.1	4.9
FIVL	4.4	3.9	4.6	3.7	3.1	3.8	3.3
SN	1.5	1.3	1.8	1.4	1.3	1.4	1.8
EN	1.6	1.4	2.4	1.5	1.5	1.8	1.9
EL	2.7	2.5	2.9	2.6	2.7	2.2	3.1
IFE	2.8	2.9	3.0	3.1	2.9	2.9	3.4
IBE	3.7	3.4	3.3	3.4	3.1	3.1	4.1
TYE	1.7	1.9	1.7	1.4	1.2	1.2	1.9
FIIIW	0.2	0.2	0.9	0.2	0.3	0.2	0.4
FIIIDW	0.3	0.3	0.3	0.3	0.3	0.3	0.4
TIVW	0.4	0.4	0.5	0.4	0.4	0.4	0.5
TIVDW	0.3	0.4	0.4	0.3	0.3	0.3	0.4
TL	9.1	8.9	8.6	9.1	8.9	9.2	10.1
TFOL	14.2	14.4	15.0	13.6	11.9	12.2	16.3
IMT	1.0	0.8	1.1	0.7	0.6	0.7	1.1

##### Holotype description.

**Figure 3.**
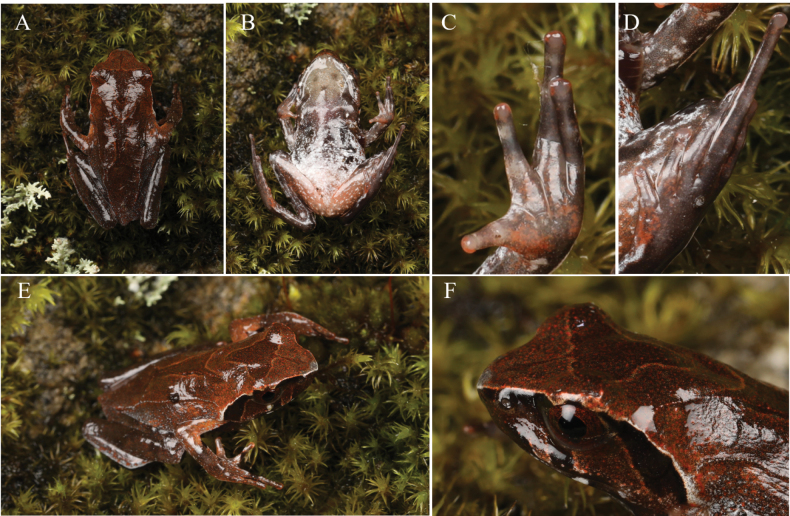
*Xenophryspangdaensis* sp. nov. in life. **A** dorsal view of body **B** ventral view of body **C** ventral view of hand **D** ventral view of foot **E** lateral view of body **F** lateral view of head.

Measurements in mm. Mature male, body slender, extremely small (SVL 21.5) (Fig. 3); small protuberance beyond cloaca from dorsal view, not visible from ventral view, not swollen; head moderate, longer than wide (HW 7.0, HL 7.6, IFE 2.8, IBE 3.7); snout nearly rounded in dorsal view, slightly protruding beyond lower jaw, angular in anterior and lateral view; loreal region acute, concave; canthus rostralis angular; dorsal surface of snout slightly concave; nostril oval, closer to eye than tip of snout (SN 1.5, EN 1.6); internarial distance greater than eyelid width, and shorter than narrowest point between upper eyelids (IN 2.5, UEW 1.9, IUE 2.9); eye diameter 1.5 × maximum diameter of visible portion of tympanum, and shorter than snout (EL 2.7, TYD 1.8, SL 3.3); eye-tympanum distance (TYE 1.7) shorter than diameter of visible portion of tympanum; tympanum oval-shaped, slightly oblique, upper border concealed by supratympanic ridge; pupil in life vertically elliptical; pineal ocellus not visible externally; vomerine ridges medium sized, orientated acutely, positioned between to slightly posterior to choanae, vomerine teeth small; maxillary teeth present; tongue moderately large, feebly notched posteriorly, medial lingual process absent.

Forelimbs moderately long and thin; forearm slender, shorter than hand (FAL 3.4, HAL 6.5); fingers moderately long, with narrow lateral fringes and rudimentary webbing (Fig. 2); finger length formula I < II < IV < III (FIL 2.6, FIIL 3.6, FIIIL 6.0, FIVL 4.4); supernumerary, thenar and metacarpal tubercles absent, with slightly visible subarticular tubercles; finger tips rounded, with subcircular pads, without terminal grooves, slightly expanded relative to digit widths (FIIIW 0.2, FIIIDW 0.3).

Hindlimbs long and thin, heels overlapping when hindlimbs held at right angles to body; thighs slightly shorter than shanks and feet (TL 9.1, SHL 10.9, FOL 10.8); toes with narrow lateral fringes, rudimentary webbing; relative toe lengths I < II < V < III < IV; toe tips rounded, with subcircular pads, terminal grooves absent; supernumerary, subarticular and outer metatarsal tubercles absent; inner metatarsal tubercles indistinct.

Skin of dorsal and ventral surfaces of head, body and limbs basically smooth; dorsal skin with very small densely-distributed granules; tympanum smooth with borders slightly raised; supratympanic ridges thin before and above departure with tympanum and gradually expanding beyond posterior edge of tympanum; skin ridges formed by small disconnected tubercles;)(-shaped skin ridge on center of dorsum, its anterior ends extending posteriorly from above tympanum; flanks with two slender skin ridges, started at the shoulder and ended on both sides of the back of the cloaca; two small pectoral glands positioned on level with axilla; femoral glands moderate, positioned posterior surface of thigh, sub-equally distant from knee and cloaca.

##### Coloration in life.

Dorsal surface basically saddle brown, darker on anterior and hindlimbs than on posterior; orange-red granules scattered on surface; dark brown) (-shaped marking with orange edge on central dorsum; ventrolateral trunk with white spots and orange dots; dark brown triangular pattern with orange edges presents between eyes, and dark brown rod-like pattern positioned in front of triangular pattern; supratympanic fold white mingled with orange flecks; temporal region under supratympanic ridge black; two dark brown patches present on upper lips under eye and nostril on side of head; eight relatively large white patches present on lower lip, symmetrically distributed; two white symmetrically curved lines on both sides of throat; many orange-red dots scattered on surface of throat; iris orange-red; two dark transverse bands on each forearm; finger tips orange-red; large white blotches on belly and ventral surfaces of hindlimbs; three dark transverse bands on anterior surface of thigh and shank; femoral glands white on thigh.

##### Coloration in preservative.

After preservation in ethanol, dorsal surface primarily brown; dark brown triangular pattern with white edges presents between eyes; brown) (-shaped marking with white edge on central dorsum; two white slender skin ridges in flanks; two dark transverse bands on each forearm; dark brown band with white dots in middle of thigh and shank; throat pale brownish grey, two white symmetrically curved lines distinct; eight distinct white patches on lower lip; chest brown with two white pectoral glands; belly pale gray-white with large black-brown blotches on sides; posterior ventral body surface, thigh, and upper part of tibia pale brown with scattered white spots; ventral surfaces of fingers and toes dark brown with white blotches.

##### Variations.

Paratypes generally resemble the holotype but with some differences. For example, a few specimens (YBU21258, YBU21262 and YBU21269) have the head width greater than the head length; YBU21258 had more and larger maxillary teeth, the tongue thinner; rod-like patterns on the top of head different between specimens. Coloration varied on ventral body, with some specimens being darker. The tips of the fingers in some specimens were not orange-red.

##### Sexual dimorphism.

Males: external vocal sac indistinct; internal vocal slit present on floor of mouth near rear of mandible, one on each side; vocal sac, vocal slits, and enlarged forearms all absent in female.

##### Tadpole.

**Figure 4.**
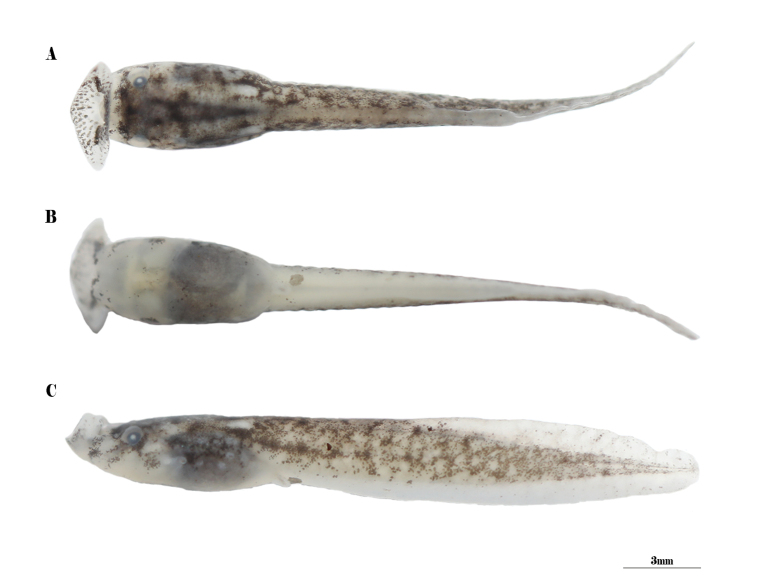
Tadpole of *Xenophryspangdaensis* sp. nov. (Gonser stage 29) from Yadong County, Tibet Autonomous Region, China. **A** dorsal view of the tadpole **B** ventral view of the tadpole **C** lateral view of the tadpole. All photographs of just- preserved specimens.

Gosner stages 25–36. Body length range from 6.3–13.8 mm (Table 4); oral disk funnel like, positioned anterior-dorsal, large, width average 1.5× (1.1–1.7, *n* = 13) maximum body width, rice-like submarginal papillae scattered on lower and upper lips and pointed towards oral cavity; nares oval and closer to eye than to snout (RN 1.0, NE 0.4); internarial distance nearly equal to interorbital distance (IND 2.8, PP 2.7); eyes dorsolateral, pupils rounded; spiracle opens left of body in dorsal view, spiracular tube positioned equidistant between tip of the snout and trunk-tail junction; the tail accounts for 0.7 of the total length (TOL 37.0, TAL 26.6); dorsal fin arise near middle of tail, upper tail fin higher than lower tail, and approximately half of tail muscle height (UF 1.5, LF 1.1, TMW 2.8)(Fig. 4).

**Table 4.** Measurements (in mm) of the tadpoles of *Xenophryspangdaensis* sp. nov. N indicates missing data. Character abbreviations are provided in the text.

Characters	1	2	3	4	5	6	7	8	9	10	11	12	13
Stage	36	31	31	31	33	31	35	32	31	27	25	29	36
BH	4.8	4.7	4.5	4.0	4.4	4.3	4.7	6.6	6.1	4.6	2.6	6.2	4.8
BL	11.4	10.3	10.8	10.4	11.0	11.5	10.9	13.8	11.8	10.4	6.3	11.9	10.6
BW	5.5	4.8	4.7	4.3	4.8	4.8	4.9	7.6	6.5	4.8	2.9	7.1	4.5
ED	0.9	1.1	1.0	1.0	1.0	1.0	1.0	1.2	1.0	0.9	0.8	1.0	1.3
IND	2.6	2.7	2.5	2.7	2.8	2.8	2.8	3.5	3.2	2.5	2.0	3.3	N
LF	1.0	1.2	1.1	1.2	1.2	1.0	1.2	1.2	1.2	1.1	0.7	1.2	1.1
NE	0.5	0.4	0.3	0.4	0.6	0.5	0.4	0.5	0.7	0.3	0.3	0.6	0.3
ODW	7.9	7.4	8.1	7.1	7.9	7.2	7.7	8.7	8.3	7.9	4.8	10.1	5.6
PP	3.1	2.7	2.5	2.8	2.8	2.5	2.9	3.3	2.9	2.6	1.6	3.3	N
RN	1.1	0.8	0.9	0.5	0.9	1.0	0.9	1.8	1.2	1.0	0.8	1.4	0.9
SS	5.8	5.8	5.6	5.7	5.7	5.4	5.7	7.4	6.8	5.4	3.7	6.4	5.3
SU	9.0	8.7	9.9	9.0	10.2	9.8	9.8	11.2	10.9	11.1	5.7	12.2	10.3
TAL	30.4	30.6	27.2	27.2	27.1	23.5	28.1	N	31.6	23.4	15.6	30.5	23.7
TMH	1.1	1.3	1.1	1.5	1.5	1.5	1.7	2.7	2.1	1.3	1.1	2.2	2.2
TMW	3.2	2.1	2.1	1.9	2.4	2.9	2.7	4.8	3.5	2.4	1.5	4.6	2.7
TOL	41.4	39.2	37.7	37.7	37.5	34.4	38.6	N	43.0	34.1	22.6	41.7	36.4
UF	1.9	1.4	1.6	1.3	1.4	1.4	1.3	2.1	1.8	1.3	0.9	1.5	1.3
TH	5.5	5.0	5.1	4.4	4.8	5.4	5.2	7.8	5.8	5.0	3.1	7.1	5.1

Coloration in preservative. Dorsal and lateral parts of body greyish white, mixed with brown patches; lateral tail semi-transparent brown, muscle scattered with many distinct brown patches; no pigment on upper and lower fins; ventral body semi-transparent white, with tiny gray pigment scattered on it, the viscera can almost be seen; lips semi-transparent white, papillae brown. Coloration in life were not noted.

##### Distribution and ecology.

**Figure 5.**
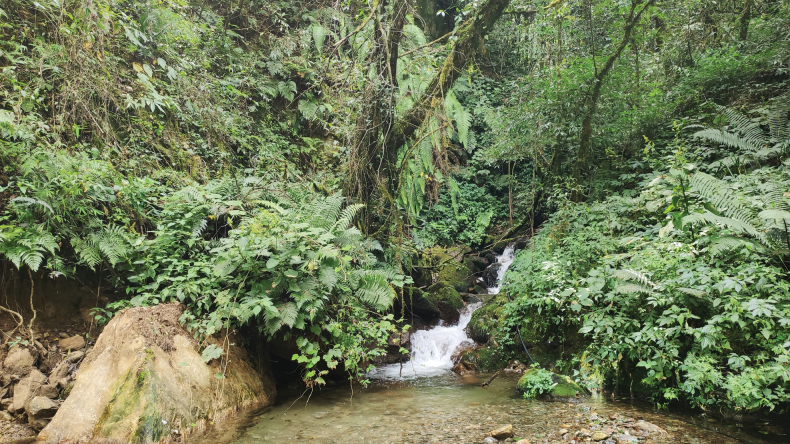
Habitat of *Xenophryspangdaensis* sp. nov. in the type locality, Pangda Village, Yadong County, Tibet Autonomous Region, China.

*Xenophryspangdaensis* sp. nov. is only known from the type locality, Yadong Town, Yadong County, Tibet Autonomous Region, China at elevations of 2003–2972 m. All calling males were recorded in August and September on ferns near or on a small stream in the tropical forest (Fig. 5). The tadpoles collected from near the type locality were from Gosner stages 25–36. The habitat is located in the small gully, both sides covered with ferns and other vegetation. None of the adults or tadpoles were found in July, and all specimens were found in late August and early September, implying that the breeding season included August and September. The sympatric species, *Raorchestesyadongensis* Zhang, Shu, Liu, Dong & Guo, 2022, *Nanoranablanfordii* (Boulenger, 1882), *Duttaphrynushimalayanus* (Günther, 1864), and *Nanoranaliebigii* (Günther, 1860) were also recorded.

##### Comparisons.

*Xenophryspangdaensis* sp. nov. is here compared with all 28 recognized species of the *Xenophrys* (Table 5). The smallest recognized species of *Xenophrys* is *X.zunhebotoensis* (male 28.4–33.9, females 37–39.5). So, by having small body size (males 18.0–22.2 mm, *n* = 6; female 23.4 mm, *n* = 1), *Xenophryspangdaensis* sp. nov. differs from all congeners from *Xenophrys*.

**Table 5.** Morphological comparison between *Xenophryspangdaensis* sp. nov. and 28 recognized species: 1. SVL in males (mm); 2. SVL in females (mm); 3. ratio TYD/EL in males; 4. ratio TYD/EL in females; 5. ratio SHL/SVL in males; 6. ratio SHL/SVL in females; 7. Horn-like tubercle at edge of upper eyelid absent (0), small (1), slightly larger (2), long point (3); 8. Vomerine teeth absent (0), present (1); 9. Vocal sac absent (0), present (1); 10. Tongue not notched (0), feebly notched(1), notched (2); 11. Lateral fringes on toes absent (0), narrow (1), wide (2); 12. Toes without webbing (0), with rudiment of webbing (1), at most one-fourth webbed (2), at least one-fourth webbed (3); 13. Subarticular tubercles on toes absent (0), indistinct (1), distinct (2); 14. Relative finger lengths; 15. Nuptial pads on finger absent (0), present (1). 16. Dorsal skin texture: smooth (0), smooth with small tubercles (1), rough (2). ? = data not available. ts= this study. Data sources: (a) Boulenger (1908), (b) Bourret (1937), (c) Bourret (1942), (d)Fei at al. (1983), (e) Ye and Fei (1992),(f) Fei et al. (1992), ((g) Huang et al. (1998), (h) Ohler et al. (2002), (i) Stuart et al. (2006), (j)Mathew and Sen (2007), (k) Fei et al. (2009), (l) Fei et al. (2012), (m) Mahony (2011), (n) Mahony et al. (2011), (o) Mahony et al. (2013), (p) Neang et al. (2013), (q)Fei and Ye (2016), (r) Deuti et al.(2017), (s) Mahony et al. (2018), (t) Shi et al. (2020), (u) Mahony et al. (2020), (v) Luong et al. (2022), (w) Lyu et al. (2023).

Species	1	2	3	4	5	6	7	8	9	10	11	12	13	14	15	16	References
*X.pangdaensis* sp. nov.	18.0–22.2	21.7–23.4	0.50–0.69	0.48–0.58	0.47–0.52	0.49–0.52	1	1	1	2	1	1	1	I<II<IV<III	0	1	t, s
*X.ancrae*	39.1–45.0	48.9	0.5–0.63	0.5–0.63	0.46–0.53	0.49	1	1	?	1	0	1	0	I<II<IV<III	1	1	o
*X.awuh*	35.7–41.1	43.7–48	0.56–0.64	0.61–0.63	0.49–0.55	0.50–0.53	0	0	1	1	0	1	0	I=II<IV<III	1	0	u
*X.auralensis*	60.1–76.7	?	0.6	?	0.51	?	1	0/1	1	0	0	1	1	II<I<IV<III	0	1	h, p
*X.damrei*	47.7–57.1	69.1	0.58	0.58	0.51	0.50	0	1	1	2	0	1	0	IV<I<II<III	0	0	n, p
*X.dzukou*	34.2–35.3	?	0.45–0.59	?	0.47–0.53	?	0	1	1	1	1	0	0	I=II<IV<III	1	0	u
*X.flavipunctata*	56.9–68.4	68–74.6	0.41–0.51	0.46–0.51	0.54–0.61	0.54–0.58	0	1	1	2	1	2	0	IV<I=II<III	1	1	s
*X.glandulosa*	76.3–81	76.5–99.5	0.51–0.65	?77	0.58–0.60	0.5–0.55	0	1	1	1	2	1	0	II=I<IV<III/ IV<II<I<III	0	0	f, g, k, l, q, s
*X.himalayana*	68–73.5	83.9	0.54	0.48	0.50–0.54	0.54	1	1	1	2	1	1	0	I<II<V<III<IV	1	1	s
*X.lekaguli*	40.1–66.6	58.6–94	0.59–0.74	0.58–0.64	0.47–0.52	0.44–0.52	1	1	0	0	0	1	0	IV<II<I<III	0	0	i, p
*X.major*	71.6–87.5	85.6–98.2	0.33–0.48	0.40–0.41	0.50–0.58	0.50–0.57	1	1	1	1	1	1	0	IV<II=I<III	1	1	q, u
*X.mangshanensis*	62.5	73	?	?	0.52	0.54	1	1	1	1	0	0	0	II<I<IV<III	0	0	f, g, k, l, q
*X.maosonensis*	58–76	68–93.5	0.5	?	0.5–0.6	?	1	1	?	1	0	2	0	?	?	0	b, c
*X.medogensis*	57.2–68	75.7–85.5	?	?	0.56	?	1	1	1	1	0	1/0	0	I<II<IV<III	0	1	d, k, l, q, t
*X.megacephala*	48.4–53.4	49.3–64.4	0.54–0.71	0.56–0.82	0.41–0.49	0.41–0.47	0	1	1	0	0	1	0	IV<II<I<III	1	1	m, u
*X.monticola*	37.8–49.1	40.5–51.1	0.38–0.59	0.44–0.71	0.45–0.51	0.46–0.51	1	0	1	1	1	1	0	I<II<IV<III	1	1	r, s
*X.numhbumaeng*	33.8–34.6	?	0.45–0.46	?	0.52–0.58	?	0	1	1	1	0	1	0	I=II<IV<III	1	0	u
*X.oreocrypta*	?	94.9	?	0.52	?	0.51	1	1	?	?	0	1	0	IV<II<I<III	?	1	s
*X.oropedion*	32.8–39.2	44.1–48.7	0.54–0.65	0.62–0.69	0.43–0.48	0.42–0.43	0	1	1	1	0	0	0	I=II=IV<III	1	1	o
*X.periosa*	71.3–93.8	112	0.44–0.58	0.45	0.51–0.58	0.51	1	1	1	?	0	1	0	IV<II<I<III	1	0	s
*X.robusta*	73.5–83.1	81.3–114	0.38–0.52	0.40–0.74	0.51–0.57	0.46–0.54	1	0	1	0	0	1	0	IV<I=II<III	1	1	a, s
*X.serchhipii*	36.1–46.7	46.1–53	0.47–0.66	0.50–0.55	0.46–0.53	0.47–0.51	0	1	1	?	0	1	0	IV<I=II<III	1	1	j, u
*X.takensis*	47.3–53	72.9	0.42–0.48	0.53	0.41–0.49	0.45	0	1	1	0	0	1	0	IV≤II<I<III/ IV=I<II<III	1	1	s, u
*X.truongsonensis*	58.8–71.4	65.6–87.3	0.51–0.67	0.53–0.59	0.55–0.64	0.54–0.58	0	1	0	1	1	1	0	I<II<IV<III	0	0	v
*X.zhangi*	32.5–37.2	?	0.5	?	0.49	?	0	1	1	1	1	0	0	II=I <IV<III	1	1	e, k, l, q
*X.zunhebotoensis*	28.4–33.9	37–39.5	0.43–0.58	0.48–0.59	0.45–0.54	0.47–0.50	0	0	1	1	0	0	0	IV<I<II≤III I≤II<IV<III	1	1	j, u
*X.pava*	36.6–42.9	41.4–52.1	0.40–0.49	0.44–0.55	0.46–0.48	0.43–0.44	0	1	1	0/1	0	0	0	II<IV<I<III	1	1	u, w
*X.dehongensis*	34.8–36.7	45.7–46.8	0.34–0.45	0.43–0.44	0.43–0.50	0.46–0.50	0	1	1	0	0	1	0	II<I<IV<III	1	2	w
*X.lancangica*	64.0–65.4	75.0–88.6	0.63–0.74	0.59–0.77	0.54–0.57	0.56–0.58	0	1	1	1	1	1	0	II<IV<I<III	1	1	w

*Xenophryspangdaensis* sp. nov. differs from *X.awuh*, *X.damrei*, *X.dzukou*, *X.flavipunctata*, *X.glandulosa*, *X.megacephala*, *X.numhbumaeng*, *X.oropedion*, *X.serchhipii*, *X.takensis*, *X.truongsonensis*, *X.zhangi*, *X.zunhebotoensis*, *X.pava*, *X.dehongensis*, *and X.lancangica* by the presence of a horn-like tubercle laterally on the upper eyelid (vs absence of horn-like tubercle at edge of upper eyelid).

*Xenophryspangdaensis* sp. nov. differs from *X.awuh*, *X.monticola*, *X.robusta*, *and X.zunhebotoensis* by presence of vomerine teeth (vs absence of vomerine teeth).

*Xenophryspangdaensis* sp. nov. differs from *X.lekaguli*, *X.truongsonensis* by presence of vocal sac (vs absence of vocal sac).

*Xenophryspangdaensis* sp. nov. differs from *X.ancrae*, *X.awuh*, *X.dzukou*, *X.flavipunctata*, *X.himalayana*, *X.major*, *X.megacephala*, *X.monticola*, *X.numhbumaeng*, *X.oropedion*, *X.periosa*, *X.robusta*, *X.serchhipii*, *X.takensis*, *X.zhangi*, *X.zunhebotoensis*, *X.pava*, *X.dehongensis* and *X.lancangica* by absence of nuptial pads on fingers (vs presence of nuptial pads on fingers).

*Xenophryspangdaensis* sp. nov. differs from *X.ancrae*, *X.awuh*, *X.dzukou*, *X.glandulosa*, *X.major*, *X.mangshanensis*, *X.maosonensis*, *X.medogensis*, *X.monticola*, *X.numhbumaeng*, *X.oropedion*, *X.truongsonensis*, *X.zhangi*, *X.zunhebotoensis*, *X.pava*, *X.lancangica* (vs tongue feebly notched), *X.auralensis*, *X.lekaguli*, *X.megacephala*, *X.robusta*, *X.takensis*, *X.dehongensis* by tongue distinctly notched (vs tongue not notched).

*Xenophryspangdaensis* sp. nov. differs from *X.ancrae*, *X.awuh*, *X.auralensis*, *X.damrei*, *X.lekaguli*, *X.mangshanensis*, *X.maosonensis*, *X.medogensis*, *X.megacephala*, *X.numhbumaeng*, *X.oreocrypta*, *X.oropedion*, *X.periosa*, *X.robusta*, *X.serchhipii*, *X.takensis*, *X.zunhebotoensis*, *X.pava*, and *X.dehongensis* by having narrow lateral fringes on toes (vs lateral fringes on toes absent), and *X.glandulosa* (vs lateral fringes on toes wide).

*Xenophryspangdaensis* sp. nov. differs from *X.dzukou*, *X.mangshanensis*, *X.oropedion*, *X.zhangi*, *X.pava*, and *X.zunhebotoensis* by toes with rudiment of webbing (vs toes without webbing), *X.flavipunctata*, and *X.maosonensis* (vs at most one-fourth webbed).

*Xenophryspangdaensis* sp. nov. differs from *X.ancrae*, *X.awuh*, *X.damrei*, *X.dzukou*, *X.flavipunctata*, *X.glandulosa*, *X.himalayana*, *X.lekaguli*, *X.major*, *X.mangshanensis*, *X.maosonensis*, *X.medogensis*, *X.megacephala*, *X.monticola*, *X.numhbumaeng*, *X.oreocrypta*, *X.oropedion*, *X.periosa*, *X.robusta*, *X.serchhipii*, *X.takensis*, *X.truongsonensis*, *X.zhangi*, *X.zunhebotoensis*, *X.pava*, *X.lancangica* and *X.dehongensis* by presence of indistinct subarticular tubercles on toes (vs absence of subarticular tubercles on toes).

*Xenophryspangdaensis* sp. nov. differs from *X.awuh*, *X.damrei*, *X.dzukou*, *X.glandulosa*, *X.lekaguli*, *X.mangshanensis*, *X.maosonensis*, *X.numhbumaeng*, *X.periosa*, and *X.truongsonensis* by dorsal skin texture smooth with small tubercles (vs dorsal skin texture smooth), *X.dehongensis* (vs dorsal skin rough).

## ﻿Discussion

The genus *Megophrys* sensu lato is a large group with extremely high species diversity. With the description of this new species, the members of the group will be 133. *Xenophryspangdaensis* sp. nov. represents the 29^th^ known species of *Xenophrys* in China and the ninth known species of the Asian horned toads from Tibet, China (Shi et al. 2020; Frost 2023). In fact, there are still some pending species whose taxonomic status needs further confirmation between Northeast India and adjacent China. Deuti et al. (2017) who described two small sized new species, *X.katabhako* and *X.sanu*, based on morphological and molecular sampling. However, Mahony et al. (2018) found that *X.katabhako* and *X.sanu* are nested within the concept of *X.monticola*. Also, similar results were obtained in this study. *X.katabhako* and *X.monticola* formed a clade. *X.sanu* and *X.zhangi* clustered into another clade. Finally, they all formed monophyletic group, but the two clades diverged considerably. For the moment, it is necessary to further investigate the taxonomic affinities of these populations by integrating more evidence. Thus, there may be some cryptic species in this group and it should be continuously paid more attentions on the species diversity of the *Megophryssensu lato*. Southern Tibet is located in the eastern of Himalayas, which is one of 36 biodiversity hotspots in the world (Basnet et al. 2019). This region is extremely unique and deserves our continued attention. In recent years, many new species have been gradually discovered in this area (Jiang et al. 2012, 2016a, 2016b, 2016c; Shi et al. 2020; Che et al. 2020; Zhang et al. 2022). There is also high species diversity in the middle and lower reaches of the Yarlung Zangbo Grand Canyon, for example, Mahony et al. (2018) revealed cryptic diversity within the *Megophrysmajor* species group, which suggests that the species diversity in this area may have been previously underestimated and therefore needs to be further investigated.

The body length (SVL) of the new species ranges from 18.0 to 22.4 mm in males and from 23.4 mm in female, however, the minimum SVL of the other recognized congeners is 28.4–33.9 mm (*X.zunhebotoensis*) in males and 37–39.5 mm (*X.zunhebotoensis*) in females. Thus, *X.pangdaensis* sp. nov. is likely to be the smallest member of all recognized species in *Xenophrys*. In addition, the members of the *Xenophrys* are very variable in body length; for example, *X.glandulosa* reaches approximately 80 mm in males and 76.5–99.5 mm in females, and the body length of *X.robusta* are even more than 100 mm in females (81.3–114.0 mm). So, it indicates that species of the same genus have a large span in body length. This also reflects the strong morphological plasticity of the Asian horned toads.

It has always been difficult to identify horned toads, especially the species with similar body length. Liu et al. (2018) recognized one sample SYSa002934 from Medog County as X.cf.pachyproctus; however, this sample clustered in a clade with *X.medogensis* in the results of Shi et al. (2020). Additionally, the evolutionary branch length in the phylogenetic tree between the two species was much shorter than between any other species, and further analysis showed that the genetic divergence between them is only 0.57% based on 16S gene. It is probable that this specimen may be misidentified previously and should be reexamined (Shi et al. 2020; this study). Furthermore, another specimen (CIB022017061805) from Bari, Medog, Tibet, China formed an independent clade in our analysis. Shi et al. (2020) treated it as M.cf.pachyproctus. So far, there is only a morphological description and no available molecular evidence from samples of the type locality (Gelin) for *J.pachyproctus*. Thus, we suggest that further sampling at Gelin would help to resolve the taxonomic problem of *J.pachyproctus* in the future.

## Supplementary Material

XML Treatment for Xenophrys pangdaensis

## ﻿Additional information

### Author ORCIDs

Guocheng Shu https://orcid.org/0000-0003-1590-6510

Ke Li https://orcid.org/0000-0001-9695-4236

Yayong Wu https://orcid.org/0000-0003-2752-4085

Qin Liu https://orcid.org/0000-0001-8402-6491

Zhongping He https://orcid.org/0009-0005-7077-8074

Lin Li https://orcid.org/0000-0002-7650-2280

He Zhang https://orcid.org/0000-0002-9511-2552

Peng Guo https://orcid.org/0000-0001-5585-292X

### Data availability

All of the data that support the findings of this study are available in the main text or Supplementary Information.

## Supplementary materials

Supplementary material 1 References utilized for morphological characters of congeners of the genus *Xenophrys* Data type xlsx https://binary.pensoft.net/file/924049This dataset is made available under the Open Database License (http://opendatacommons.org/licenses/odbl/1.0/). The Open Database License (ODbL) is a license agreement intended to allow users to freely share, modify, and use this Dataset while maintaining this same freedom for others, provided that the original source and author(s) are credited.Guocheng Shu, Ke Li, Yayong Wu, Qin Liu, Zhongping He, Ling Li, He Zhang, Peng Guo
